# Examining the utility of digital phenotyping for the prediction of intrusive experiences

**DOI:** 10.1080/20008066.2026.2646126

**Published:** 2026-05-06

**Authors:** Tomas Meaney, Vijay Yadav, Isaac Galatzer-Levy, Richard Bryant

**Affiliations:** a School of Psychology, University of New South Wales, Sydney, Australia; b Department of Psychiatry, NYU Grossman School of Medicine, New York University, New York, NY, USA; c Black Dog Institute, Sydney, Australia

**Keywords:** Post-traumatic stress disorder, intrusions, digital phenotyping, emotion, machine learning, Trastorno por estrés postraumático, intrusiones, fenotipificación digital, emoción, aprendizaje automático

## Abstract

**Background:** Intrusive experiences related to witnessing a traumatic event are the core symptom of post-traumatic stress disorder (PTSD), and have been shown to be predicted by peritraumatic emotional arousal. However, research into the role of peritraumatic arousal in the development of intrusions has been limited by a reliance on self-report scales.

**Objective:** This study aimed to examine whether facial, focal, and language phenotypes of peritraumatic emotional arousal could also contribute to the prediction of intrusive experiences.

**Method:** This longitudinal study recruited university students (*N* = 81), who were recorded describing their response to an analogue trauma (car accident video). Participants’ facial, vocal, and linguistic features were extracted from these recordings, as well as their self-reported emotional experiences, and input into machine learning (random forest regression) models to predict whether they would have intrusive experiences of the analogue trauma three days after their exposure to it.

**Results:** Random forest regression models were able to predict variance in responsiveness to triggers that reminded individuals of the analogue trauma (*R*^2^ = 0.23) and whether they had vivid memories of the analogue trauma (*R*^2^ = 0.55). Vocal and language features made the greatest contribution to the prediction of responsiveness to triggers, while initial self-reported memory vividness made the greatest contribution to the prediction of three-day memory vividness.

**Conclusions:** These findings suggest that facial, vocal, and language phenotypes, in combination with self-report measures, can have utility for predicting the occurrence of specific intrusive experiences following exposure to an analogue trauma in a university sample. Further research into this approach in clinical samples is required to demonstrate its utility for predicting intrusions in those with PTSD.

## HIGHLIGHTS

- This study examined whether facial, vocal, and language features extracted from recordings of exposure to an analogue trauma could be used to predict intrusive experiences.
- Variance in responsiveness to trauma reminders after exposure to the analogue trauma was predicted by a machine learning model that primarily utilised vocal and language features.
- Variance in mean vividness of memories of the analogue trauma was predicted by a machine learning model that relied on self-reported vividness.

## Abstract (es)

**Antecedentes:** Las experiencias intrusivas relacionadas con presenciar un evento traumático son el síntoma central del trastorno por estrés postraumático (TEPT) y se ha demostrado que pueden predecirse por la activación emocional peritraumática. Sin embargo, la investigación sobre el papel de la activación peritraumática en el desarrollo de intrusiones se ha visto limitada por la dependencia de escalas de autoinforme.

**Objetivo:** Este estudio tuvo como objetivo examinar si los fenotipos faciales, focales y lingüísticos de la activación emocional peritraumática también podrían contribuir a la predicción de experiencias intrusivas.

**Método:** Este estudio longitudinal reclutó estudiantes universitarios (*N* = 81), quienes fueron grabados describiendo su respuesta a un trauma análogo (video de accidente automovilístico). Se extrajeron de estas grabaciones características faciales, vocales y lingüísticas, así como sus experiencias emocionales auto-reportadas, y se introdujeron en modelos de aprendizaje automático (regresión de bosque aleatorio) para predecir si tendrían experiencias intrusivas del trauma análogo tres días después de la exposición.

**Resultados:** Los modelos de regresión de bosque aleatorio pudieron predecir la varianza en la reactividad a disparadores que recordaban a los individuos el trauma análogo (*R*^2^ = 0.23) y si tenían recuerdos vívidos del trauma análogo (*R*^2^ = 0.55). Las características vocales y lingüísticas realizaron la mayor contribución a la predicción de la reactividad a disparadores, mientras que la vividez de la memoria inicial auto-reportada aportó la mayor contribución a la predicción de la vividez de la memoria a los tres días.

**Conclusiones:** Estos hallazgos sugieren que los fenotipos faciales, vocales y lingüísticos, en combinación con medidas de autoinforme, pueden tener utilidad para predecir la ocurrencia de experiencias intrusivas específicas tras la exposición a un trauma análogo en una muestra universitaria. Se requiere más investigación sobre este enfoque en muestras clínicas para demostrar su utilidad en la predicción de intrusiones en personas con TEPT.

## 1. Introduction

Emotionally evocative intrusive memories of a traumatic event, typically involving sensory aspects of the memory, are a central feature of post-traumatic stress disorder (PTSD) (American Psychiatric Association, 2022). Given the negative psychological consequences associated with intrusions (Ehlers et al., 2004), researchers have been interested in trying to identify factors that predict their development. The dual representation theory of PTSD claims that the intensity of peritraumatic sensory and affective experiences during the time of a traumatic event means that they are strongly encoded, while contextual details of the event are less strongly encoded, resulting in intrusive experiences that feel ‘real’ and are emotionally distressing (Brewin & Burgess, 2014). This theory has been supported by findings that the intensity of emotional experience at the time of the traumatic event is a strong predictor of PTSD intrusion symptoms in retrospective clinical research studies (O’Donnell et al., 2010; Ozer et al., 2003). Intense levels of peritraumatic emotional arousal have also been shown to contribute to the development of intrusions in longitudinal studies (Doron-LaMarca et al., 2015; Marshall et al., 2006). Although naturalistic studies of trauma survivors allow inferences to be drawn about the relationship between initial emotional reactions and the development of intrusions, they are limited by their reliance on retrospective reports.

The trauma film paradigm is a methodology which permits a more immediate assessment of initial responses to a stressful stimulus and the relationship of these responses with subsequent intrusion experiences, thereby addressing some of the limitations associated with using retrospective reports to measure peritraumatic response (Holmes & Bourne, 2008). This paradigm involves participants watching a video of a traumatic incident, such as the aftermath of a car accident, reporting on their emotional reactions to the video, and then completing a diary in which they record the occurrence of intrusions over a disclosed period of time (Holmes et al., 2004; Rattel et al., 2019; Stuart et al., 2006). An individual participant data meta-analysis of 16 studies using this paradigm found that an initially lower self-reported emotional response to traumatic film footage was associated with an absence of intrusions in the week after watching the film (Clark et al., 2015), consistent with peritraumatic arousal findings in clinical samples. However, such research has depended on self-report scales to index participants’ emotional states after watching trauma films, which are prone to a range of biases, such as social desirability responding (van de Mortel, 2020).

A novel approach for measuring peritraumatic emotional experience to predict intrusions beyond self-report measures is to analyse specific facial, vocal, and linguistic phenotypes of emotion. Such phenotypes represent another way to measure emotional experience, in a way that is more behaviourally and biologically based. This approach is consistent with basic emotion theory, which proposes that non-verbal emotion expressions represent coherent patterns of emotional response that are consistent with subjective emotion experiences and signal the emotional state of the individual expressing them (Keltner et al., 2019; Shariff & Tracy, 2011). Accounting for a range of channels of emotion response aims to simulate the way in which clinicians integrate facial, vocal, and language features to inform their assessment of patients (Schultebraucks & Galatzer-Levy, 2019). Consistent with clinical presentations of PTSD, decreased positive affect and increased negative affect expression in the face, as well as specific vocal and language features, have been linked with symptoms of PTSD (Blechert et al., 2013; Kirsch & Brunnhuber, 2007; Scherer et al., 2013). Such digital phenotypes, extracted from recordings taken shortly after exposure to a trauma, have been incorporated into a neural network model to accurately discriminate PTSD status (Schultebraucks et al., 2022). Language (e.g. first-person pronouns), vocal (e.g. mean volume), and facial features (e.g. fear and anger facial expressivity) were found to contribute substantially to the performance of this classification model. It is expected that since these digital phenotypes have been shown to relate to PTSD, and as they are designed to index emotional experience and arousal (Bantum & Owen, 2009; Burris et al., 2014: Hutto & Gilbert, 2014: Venkatesan et al., 2023), which at the time of the event has been shown to contribute to the development of intrusions (Doron-LaMarca et al., 2015; Marshall et al., 2006), these facial, vocal, and language phenotypes could also contribute to the prediction of intrusive experiences following exposure to an analogue trauma.

The present study aimed to determine whether digital phenotypes, in combination with self-report measures, could be used to predict the occurrence of intrusive experiences using the trauma film paradigm. Based on the reviewed research, we hypothesized that if integrated into random forest regression models, digital phenotype variables extracted from participant responses to an analogue trauma (car accident video) could predict whether participants had memories, images, thoughts, responses to reminders of the video, vivid memories, and strong emotions in the week after it was viewed. However, as analogue trauma research has primarily used self-report measures of emotional arousal to predict intrusive experiences, this study will examine the relative contribution of self-report and digital phenotype measures to the prediction of intrusions. Given the centrality of intrusions to PTSD, it was hypothesized that the facial, vocal, and linguistic features that were predictive of PTSD in previous digital phenotype studies (Schultebraucks et al., 2022) would contribute to the predictive capacity of machine learning models in this study. However, these hypotheses were made tentatively, given that this kind of digital phenotyping approach has not been used in an analogue trauma design with a non-clinical sample.

## 2. Methods

The data collected in this study consisted of responses to self-report questionnaires and recordings of participants’ facial and verbal responses to an analogue trauma (car accident video). All analysed deidentified data from this study are available in an OSF repository (Meaney, 2025). Data analyses were run in R version 4.3.1 (R Core Team, 2021) and in the Scikit-learn package (Pedregosa et al., 2011) in Python. The data-analysis code used in this study is available upon request. We report how we determined our sample size, all data exclusions, all manipulations, and all measures in the study. All procedures for the study were approved by the University of New South Wales Human Research Ethics Committee (case 3549).

### 2.1. Participants

Participants in this study were 102 students from the University of New South Wales, Sydney, Australia, who were either recruited as part of standard research participation or paid for their participation, making it a local convenience sample. This sample size was determined based on the requirements of random forest regression models trained and tested within nested cross-validation pipelines (Vabalas et al., 2019; Varma & Simon, 2006). An informed consent sheet in Qualtrics was completed by each participant. The exclusion criteria for the study were scoring in the severe range on any of the subscales of the 21-item Depression, Anxiety, and Stress Scale (DASS-21) (Lovibond & Lovibond, 1995), being under 18 years of age, and previously having been involved in a motor vehicle accident. Four participants were excluded because of technical difficulties in the recording of their responses, five opted out of viewing the car accident video, seven were excluded for scoring above the specified threshold on the DASS-21, and five were excluded for having excessive missing data (over 50%) on their diary measure of intrusions. This resulted in a final sample of 81 participants eligible for the analysis. Most of the sample identified their sex at birth as female (58%), and the mean age of participants was 20.68 years (*SD* = 3.54). Our sample size was larger than those in previous studies examining the digital phenotypes associated with symptoms of PTSD (Schultebraucks et al., 2022).

### 2.2. Materials

#### 2.2.1. Negative video

Participants were asked to view a six-minute video depicting the aftermath of a serious car accident involving multiple injured people and one deceased individual. This is a standard video that has been used in the context of prior intrusion studies (Stuart et al., 2006). A one-minute recording of the participant describing their reaction to the video was taken immediately after the video ended. The video recording was taken at a frame rate of 60 frames per second using the QuickTime application.

#### 2.2.2. Screening measure

The DASS-21 (Lovibond & Lovibond, 1995) is a 21-item self-report questionnaire, which is designed to assess depression, anxiety, and stress. It was used to screen out psychologically distressed participants to preclude them from viewing the unpleasant images. Participants were also asked to provide their age and sex at birth.

#### 2.2.3. Digital phenotypes

Participants’ reactions to the car accident video were analysed in terms of facial, vocal, and linguistic phenotypes using OpenWillis (Worthington et al., 2024) and the Linguistic Inquiry and Word Count – version 22 (LIWC-22) (Boyd et al., 2022) software.

OpenWillis uses DeepFace to measure framewise the intensity of Facial Action Coding System (FACS) (Ekman & Friesen, 1978) units on a range of −1 (expressivity of that emotion below baseline) to 1 (expressivity of that emotion above baseline) to produce facial emotion expressivity scores. FACS units index the intensity of activity in both individual and groups of muscles in the face (which each have their own Facial Action Unit) that have been linked with the six primary emotions: sadness, fear, surprise, anger, disgust, and fear (Ekman & Friesen, 1978). DeepFace has been shown to be 97% accurate in the identification of facial landmarks on which it has been previously trained (Taigman et al., 2014) and to have 94% accuracy in identifying human emotions (Venkatesan et al., 2023).

Parselmouth is the package in OpenWillis that is used to measure vocal variables (Jadoul et al., 2018). Parselmouth is an implementation of the Praat software library for Python (Boersma & Weenink, 2024), which produces vocal variables such as volume, mean fundamental frequency (‘f0_mean’) (see Supplementary Material for a glossary that includes the OpenWillis and LIWC-22 variables), and deviation in fundamental frequency. It also measures and produces more complex vocal features, such as CPP (cepstral peak prominence; ‘cpp_var’) and the variance in MFCCs (mel-frequency cepstral coefficients, e.g. ‘mfcc12_var’). Praat software has been demonstrated to have good convergent validity (with other vocal software) and reliability in the identification of vocal features (Burris et al., 2014). OpenWillis uses WhisperX to convert audio into text, so that language analysis can be performed. WhisperX has a word error rate of 9.7%, which is an improvement on past speech-to-text models (Bain et al., 2023). The Valence Aware Dictionary and sEntiment Reasoner (VADER) (Hutto & Gilbert, 2014) analysed the language sentiment of the extracted text using a rule-based algorithm that produces mean scores from −1 (negative sentiment) to 1 (positive sentiment) in terms of the emotional valence of the text.

LIWC-22 library software (Boyd et al., 2022) was used to count the words in the extracted text to provide scores for the number of words used for each of its dictionaries (e.g. score for number of ‘Affect’ words). LIWC software has been demonstrated to have higher discriminant and convergent validity with measures of emotion (observer rating and self-report measures) than other text analysis software (Bantum & Owen, 2009).

#### 2.2.4. Affect scale

Participants rated how negatively they felt after viewing the car accident video on a 10-point visual analogue scale (1 = not at all negative; 10 = extremely negative).

#### 2.2.5. Intrusion measures

Six intrusion measures were given to participants three minutes after the car accident video, and at three time-points each day for seven days after they viewed it. These intrusion measures were partially derived from the Intrusive Memories Questionnaire (IMQ) (Baker & Goodson, 2023), but other, more specific, measures were added based on their relevance to important aspects of intrusive experiences associated with PTSD, such as vividness, imagery, and emotional intensity (Clark et al., 2015; Doron-LaMarca et al., 2015; Morina et al., 2013). Each measure indexed a separate component of intrusions: experience of memories, response to triggers, experience of images, experience of emotions, and vividness of the intrusions. In this same order, each of the measures asked how true it was that: ‘Memories of the video popped into my mind’, ‘Other things kept making me think of the video’, ‘I thought about the video when I didn’t mean to’, ‘It was difficult to get the images from the video out of my mind’, ‘I had waves of strong feelings about the video’, and ‘My memory of the video is very vivid’ (1 = not at all true; 5 = extremely true). Mean scores for participant responses to these questions at the three time-points each day for seven days were defined as the following six intrusion measures: Memories, Triggers, Thoughts, Images, Emotions, and Vividness. These six measures had ‘good’ to ‘excellent’ levels of internal consistency (*α* = .89), indicating that they were indexing the same underlying construct of intrusive experiences.

### 2.3. Procedure

Participants were shown a video depicting the aftermath of a car accident over Microsoft Teams. Participants were then recorded using QuickTime providing a one-minute description of their reaction to the video. Three minutes after providing this response, they were given the six intrusion measure statements and asked how true these were for them at that time, then instructed on how to download and use the Smartphone Ecological Momentary Assessment (SEMA-3) phone application, which sent participants the intrusion questions at three time-points each day (10 a.m., 2 p.m., and 6 p.m.).

### 2.4. Data analysis

Using R Statistical Software Version 4.3.1 (R Core Team, 2021) and the Scikit-learn package (Pedregosa et al., 2011) in Python, we evaluated the estimated capacity of random forest regression models, built with all the facial, vocal, and language variables extracted (see Supplementary Material for a glossary of the variables incorporated into the random forest regression models) from participant recordings, and the relevant intrusion measures taken three minutes after participants were exposed to the car accident video, to predict mean scores on the six intrusion measures. No other measures were included in the random forest regression models. The six intrusion measure scores were originally planned to be analysed in terms of mean scores across the seven days in which participants were asked to respond to the questions. However, from day 4 onwards, the number of missing entries increased substantially, affecting the extent to which the mean scores would be based on real, as opposed to imputed scores. As such, it was determined that mean intrusion scores would be based on participant scores across the first three days. Across the three days, participants were retained in the analysis if they had responded to over 50% of the SEMA-3 questions. A random forest-based package in R Statistical Software called MissForest was used to impute the missing scores from participants who were retained, given its established efficacy for dealing with data that is non-linear and has high dimensionality (Stekhoven & Bühlmann, 2012).

Once the data had been imputed, and mean values had been calculated for the intrusion measures across the three days after the car accident video, a nested cross-validation approach was used to build and estimate the performance of machine learning (random forest regression) models to predict variance in the mean intrusion measures. The random forest method was chosen given its established performance in accurately predicting variance relative to other machine learning methods (e.g. regularized regression), robustness against overfitting, and minimal hyperparameter tuning requirements (Chen et al., 2020; Gregorutti et al., 2017). The recursive feature elimination (RFE) method was used to reduce the number of features in the random forest model, as it has been shown to improve the performance of random forest models, given its capacity to account for interactions between complex sets of features (Gregorutti et al., 2017). The scaling of the predictors, feature selection with RFE, and random forest model training were completed within the inner folds of a nested cross-validation pipeline, to ensure that feature scaling and selection did not leak information from the training set to the test set, thereby minimizing bias. Nested cross-validation is considered a suitable alternative to evaluating model performance on a test sample that is independent from feature selection and model training, as the estimates of model performance have been shown to closely correspond with actual model performance with external test sets (Vabalas et al., 2019; Varma & Simon, 2006).

**Figure 1.**
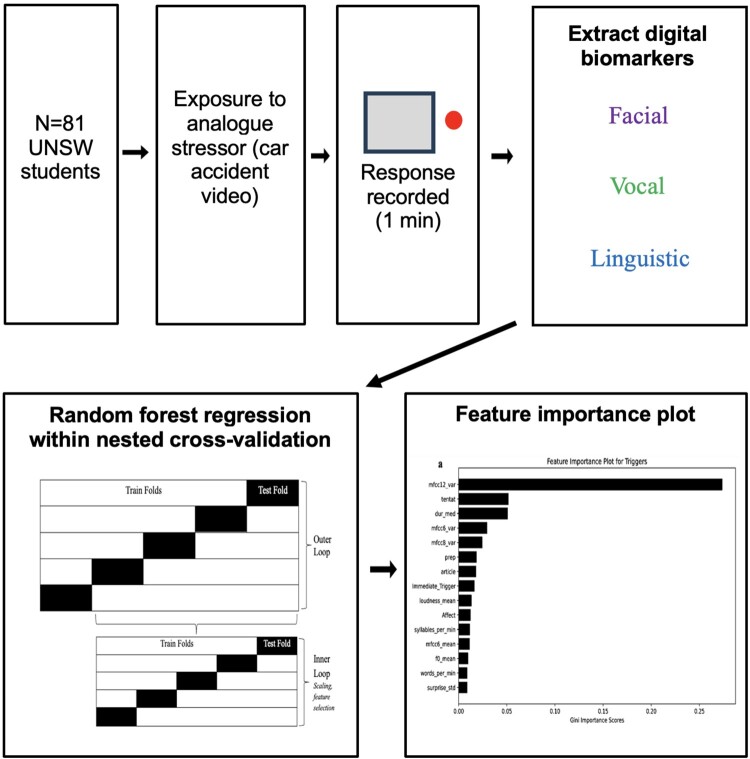
Experimental process. UNSW = University of New South Wales.

The features that minimized the root mean square error (RMSE) were selected and retained in a ‘best model’ for predicting variance in each intrusion measure. RMSE is the square root of the mean squared error (MSE) of the difference between the actual and predicted scores on the target variables (intrusion measures). The estimated generalized performance of this model was evaluated across the outer five folds using RMSE, mean absolute error (MAE), and *R*^2^, providing average RMSE, MAE, and *R*^2^ scores. MAE refers to the average absolute difference between actual and predicted values for the target variables, while *R*^2^ is a measure of how much variation in the target variable (intrusion measures) is captured by the model using the predictor variables (digital phenotype variables and immediate self-report measures). Feature importance scores were calculated for each of the predictor variables based on the average decrease in Gini impurity that they produced across all decision trees in the random forest, as this approach has been shown to have an enhanced capacity to accurately represent feature importance over other methods, such as Shapley Additive exPlanations (SHAP) importance scores (Wang et al., 2024). These Gini importance scores for each feature were used to create feature importance plots for the random forest regression models corresponding to each intrusion measure. The entire experimental process is represented in Figure 1.

## 3. Results

### 3.1. Random forest regression models

The random forest regression model for predicting whether participants would have memories of the video (Memories) over the three days after they watched it had an average *R*^2^ of −0.09 and used 199 features. The random forest regression model for predicting variance in whether other things would remind participants of the video (Triggers) over the three days after they watched it had an average *R*^2^ of 0.23 and used 201 features. The random forest regression model for predicting variance in whether participants thought about the video when they did not mean to (Thoughts) over the three days after they watched it had an average *R*^2^ of −0.18 and used 198 features. The random forest regression model for predicting variance in whether participants saw images of the video (Images) over the three days after they watched it had an average *R*^2^ of −0.13 and used 201 features. The random forest regression model for predicting variance in whether participants felt strong emotions about the video (Emotions) over the three days after they watched it had an average *R*^2^ of −0.21 and used 198 features. The random forest regression model for predicting variance in whether participants’ memory of the video was very vivid (Vividness) over the three days after they watched it had an average *R*^2^ of 0.55 and used 198 features. Table 1 depicts the estimated random forest regression performance metrics for each of the intrusion measures.

**Table 1.** Performance metrics for the random forest regression models.

Measures	RMSE	MAE	*R*^2^
Memories	0.66	0.51	−0.09
Triggers	0.61	0.49	0.23
Thoughts	0.74	0.60	−0.18
Images	0.71	0.54	−0.13
Emotions	0.78	0.61	−0.21
Vividness	0.81	0.64	0.55

Note: RMSE = root mean square error; MAE = mean absolute error.

### 3.2. Digital phenotype feature importance

**Figure 2.**
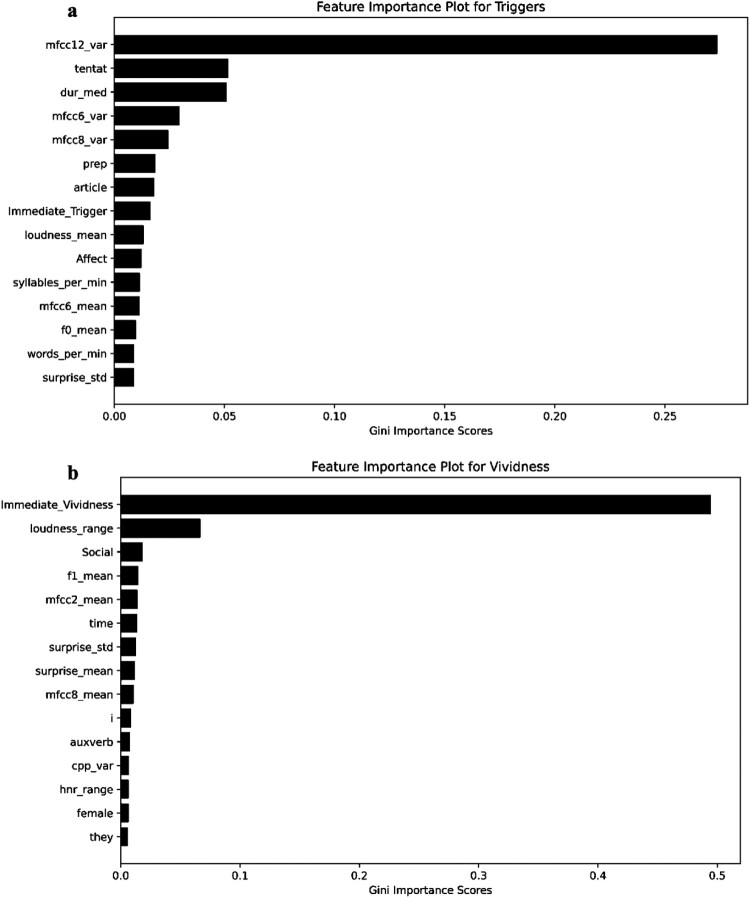
Feature importance plots for the random forest regression models for (a) triggers and (b) vividness.

Figure 2 displays feature importance plots for the digital phenotype variables that were important for predicting variance in the mean scores for intrusion variables (Triggers and Vividness). Audio (primarily ‘mfcc12_var’, but also ‘dur_med’ and ‘mfcc6_var’) and language (such as ‘tentat’ and ‘prep’) variables contributed to the estimated capacity of the random forest regression models to predict variance in the mean likelihood of response to triggers in the three days after the car accident video. Participants’ self-report of the vividness of the images seen by them three minutes after watching the video was the most important variable for the estimated capacity of the random forest regression model to predict variance in the vividness of their memories of the car accident video. Audio (‘loudness_mean’) and language (‘Social’) variables were also relevant features in this model, but to a lesser extent than the self-report variable.

## 4. Discussion

The current study examined the estimated capacity of random forest regression models, built with digital phenotype and self-report variables taken at the time of exposure to an analogue trauma (car accident video), to predict variance in measures of intrusions in the three days after exposure. Such a model, tuned in a nested cross-validation pipeline, was estimated to be able to predict the variance in whether participants reacted to triggers of the analogue trauma in the three days after they viewed it by an ‘acceptable’ amount (Chicco et al., 2021). Reactivity to reminders being able to be predicted by measures of emotional experience/distress taken during encoding is consistent with past findings from analogue trauma studies (Clark et al., 2015; Rattel et al., 2019).

The variables that were important for the estimated prediction of variance in whether participants responded to reminders of the video were specific vocal and language variables, not the self-report measure that was taken three minutes after they viewed the video, demonstrating the value of using digital phenotypes to predict this intrusive experience. The MFCC variables found to be important for prediction in variance of these measures have previously been linked to psychological distress (Taguchi et al., 2018), supporting the dual representation theory of intrusions that peritraumatic emotional distress is associated with continued intrusive experiences and emotional responsivity (Brewin & Burgess, 2014). It also supports basic emotion theory (Keltner et al., 2019) in finding convergence and coherence between measures of non-verbal emotion expression and subjective self-report ratings of emotional reactivity. The relevance of these particular vocal and language variables also supports our hypothesis that the digital phenotypes that are most relevant to the estimated prediction of intrusion measures would be similar to those that were important for predicting scores on measures of PTSD (Blechert et al., 2013; Kirsch & Brunnhuber, 2007; Scherer et al., 2013; Schultebraucks et al., 2022). Responses to triggers of past traumatic incidents are often accompanied by increases in physiological arousal and changes in behaviour (O’Donnell et al., 2010; Ozer et al., 2003), which could explain why variance in this intrusion measure was estimated to be able to be predicted by random forest models built with digital phenotypes that aim to measure peritraumatic emotional arousal.

The mean of whether participants’ memory of the analogue trauma was vivid in the three days after watching it was estimated to be predicted by a random forest regression model to a ‘good’ degree (Chicco et al., 2021). Facial, vocal, and linguistic variables made a much smaller contribution to model performance than the self-reported vividness of the memories experienced in the three minutes after participants watched the video, which could be explained by reported vivid mental imagery not having a direct relationship to physiologically detectable emotional responses. This would be consistent with findings that the experience of vivid intrusions after exposure to an analogue trauma is related to the characterological self-reported vividness of mental imagery that participants experience generally, rather than their peritraumatic emotional response (Morina et al., 2013). These findings support the notion that self-report measures of individuals’ experiences of vivid mental imagery may be the most effective means by which to predict this aspect of the experience of intrusions.

Whether participants had memories, images, thoughts, and feelings related to the car accident video across the three days after they viewed it was not estimated to be able to be predicted by the random forest models built with digital phenotypes. This stands in contrast to past findings in which peritraumatic emotional experiences were related to intrusive experiences (Doron-LaMarca et al., 2015; Holmes et al., 2004; Marshall et al., 2006). There are several possible explanations for why these models were estimated to be unable to predict variance in the target intrusion measures. Given that this study was conducted in a non-clinical sample and only used a brief distressing video, it is possible that the distress experienced by participants was not sufficient to meaningfully elicit intrusive experiences for long enough, as in previous studies (Rattel et al., 2019), or there may have been too much noise introduced by extraneous phenotype variables that RFE was not able to eliminate. Alternatively, this digital phenotyping approach may not be sensitive enough to longitudinally predict intrusive experiences. Further research using this digital phenotype analysis approach in clinical PTSD populations is needed to establish whether the encoding of PTSD intrusions can be predicted by variance in peritraumatic emotional expression.

The current study had several limitations. The first was the need to impute missing data from the intrusion measures collected across the three days after participants viewed the video. This meant that the results from this study were partly based on data that were mathematically generated, although these imputations were based on collected data and used a well-validated imputation method (Stekhoven & Bühlmann, 2012). Another limitation is that this study examined the clinical construct of intrusions in a non-clinical population. This limits the degree to which these findings can be extrapolated to the experience of intrusions for individuals with PTSD, a limitation that can be addressed by testing this approach in clinical PTSD populations. It also impacts the intensity of intrusions likely to have been experienced by these non-clinical participants, and subsequently the capacity of the models to predict intrusion experiences. A further limitation is that the data were only analysed in terms of the mean scores of participants on the intrusion measures across the three days after exposure to the analogue trauma. Trajectory analysis is an alternative approach that could have better captured the complexity of such longitudinal data and would be a valuable avenue for future research in this area. The generalizability of this study’s results to clinical experiences of intrusions is also limited by its non-clinical sample.

## 5. Conclusions and implications

This study examined whether machine learning (random forest regression) models built with digital phenotype variables would be estimated to predict variance in whether individuals had intrusive experiences in the three days after experiencing an analogue trauma (car accident video). Such models were estimated to be able to predict some of the variance in the response to triggers that reminded participants of the video and whether they had vivid memories of it. While immediate self-reported vividness of the memories of the car accident video was the most important variable for the estimated prediction of vividness of the memory across the three days, vocal and language variables were most important for the estimated prediction of variance in response to triggers, illustrating the value of examining multiple channels of emotion response to identify those that contribute most to predicting specific intrusive experiences. These findings suggest that digital phenotyping of peritraumatic emotional experience could have utility for predicting responsiveness to reminders of an analogue trauma. Following further evaluation of this approach in clinical populations, this could allow for the development of a digital phenotype profile that could be used to identify those at greater risk of being responsive to triggers and reminders of a traumatic event that they have recently experienced (e.g. individuals assessed in emergency rooms). Ultimately, as this approach can be deployed and used so quickly and efficiently, it could contribute to these individuals receiving a more timely and immediate debriefing about their experiences and provision of treatment resources, leading to an overall improvement in the efficiency of treatment allocation for those who are more responsive to traumatic reminders. The further evaluation of this approach in clinical settings and populations is a necessary next step in determining its utility for predicting intrusive experiences in those with PTSD, and eventually improving treatment delivery.

## Supplementary Material

Supplementary Material anonymous.docx

## Data Availability

The deidentified variable datasets analysed during this study are available in the OSF repository: https://osf.io/szdwn/overview?view_only=1e02516672e745b38d61cc05156af380. The raw video-recording data analysed during this study are not publicly available owing to privacy concerns and ethical requirements.

## Open scholarship

This article has earned the Center for Open Science badges for Open Data and Open Materials through Open Practices Disclosure. The data and materials are openly accessible at https://openwillis.brooklyn.health/15883a8fe04780739400c1d8ad94bb39?v=15883a8fe047806aa291000cb85dceae and https://osf.io/szdwn/overview?view_only=1e02516672e745b38d61cc05156af380.
