# Supplementary material for: Examining the utility of digital phenotyping for the prediction of intrusive experiences
Source: Eur J Psychotraumatol. 2026 May 6;17(1):2646126. doi: 10.1080/20008066.2026.2646126 (PMC13151764; doi:10.1080/20008066.2026.2646126)
Supplement: Supplementary Material anonymous.docx [file ZEPT_A_2646126_SM3979.docx]

**Supplementary Material**


**Glossary of Variables Used in Random Forest Regressions**

*Self-Report Intrusion Measures (administered 3-minutes after exposure)*

For each of the following statements, please respond on the corresponding scale for how true each is from 1 = not at all true to 5 = extremely true.

Immediate_Memories: “Memories of the video popped into my mind”

Immediate_Trigger: “Other things kept making me think of the video”

Immediate_ Thoughts: “I thought about the video when I didn’t mean to”

Immediate_Images: “It was difficult to get the images from the video out of my mind”

Immediate_Emotions: “I had waves of strong feelings about the video”

Immediate_Vividness: “My memory of the video is very vivid”

*OpenWillis Variables*

overall Overall framewise displacement in facial landmark coordinates (mean (corresponds to “Overall Facial Activity”) and std (standard deviation))

upper_face Framewise displacement in facial landmark coordinates in the upper half of the face (mean and std)

lower_face Framewise displacement in facial landmark coordinates in the lower half of the face (mean and std)

mouth_openness Ratio of mouth height by the minimum of the lower lip and upper lip height (mean and std)

eyebrows Expressivity of eyebrows in terms of average Euclidean movement at this landmark (mean and std)

lips Expressivity of lips in terms of average Euclidean distance movement at this landmark (mean and std)

angry Action unit activation related to the emotion of anger (mean and std)

disgust Action unit activation related to the emotion of disgust (mean and std)

fear Action unit activation related to the emotion of fear (mean and std)

happiness Action unit activation related to the emotion of happiness (mean (corresponds to “Facial Happiness”) and std)

sadness Action unit activation related to the emotion of sadness (mean (corresponds to “Facial Sadness”) and std)

surprise Action unit activation related to the emotion of surprise (mean and std)

neutral Action unit activation related to the experience of neutral emotion (mean and std)

composite Total action unit activation

blinks Count of the number of eye blinks

blink_rate Number of blinks per minute

f0 Fundamental frequency of voice in Hertz (mean (corresponds to “Mean Vocal Pitch”), std (corresponds to “Vocal Pitch Deviation”) and range)

f1-4 Formant frequencies f1-4 of voice in Hertz (mean, std and range)

loudness Volume of voice in decibels (mean (corresponds to “Mean Vocal Volume”), std and range)

hnr Harmonics-to-noise ratio (mean, std and range)

jitter Measure of the perturbation in the fundamental frequency of voice (corresponds to “Jitter”)

jitter_abs Absolute measure of the perturbation in the fundamental frequency of voice

jitter_rap Relative average perturbation in voice

jitter_ppq5 Five-point period perturbation quotient of voice

jitter_ddp Measure of the average absolute difference between the successive differences between consecutive intervals of speech divided by the average interval

shimmer Average absolute difference between amplitudes of two consecutive periods of speech

shimmer_db Measures the average absolute difference of the base 10 log of the difference between two successive periods of speech (corresponds to “Shimmer”)

shimmer_apq3 Quotient of amplitude disturbance within three periods of speech

shimmer_apq5 Quotient of amplitude disturbance within five periods of speech

shimmer_apq11 Quotient of amplitude disturbance within eleven periods of speech

shimmer_dda Average absolute difference between successive differences in amplitudes across a number of periods

gne_ratio Glottal-to-noise excitation ratio

mfcc1-14 The first 14 Mel-Frequency Cepstral Coefficients (MFCCs), which measure the short-term power spectrum of vocal production, derived from a linear cosine transform of a log power spectrum on a nonlinear mel scale of frequency (mean and var(variance)

cpp Cepstral peak prominence, which measures dysphonia and breathiness (mean and var)

spir Number of pauses (longer than 50 milliseconds and shorter than 2 seconds) in proportion to total speech

dur_med Mediation duration of silences (longer than 50 milliseconds and shorter than 2 seconds)

dur_mad Median absolute deviation of silence duration (longer than 50 milliseconds and shorter than 2 seconds)

silence_ratio The percentage of frames with no voice (corresponds to “Percent of Frames with Silence”)

rel_fo_sd Pitch variation as measured by standard deviation of F0 contour of vocal segments longer 100 milliseconds relative to its mean

rel_se0_sd Speech loudness variation as measured by the standard deviation of energy of vocal segments longer than 100 milliseconds relative to its mean

words_per_min The number of words used per minute

syllables_per_min The number of syllables used per minute

mean_pre_word_pause Mean pause time before word use in seconds

mean_pause_variability Mean variability in pauses in speech

speech_percentage Time participants was speaking over total time of recording

sentiment_pos Measure of positive valence of speech ranging from 0-1 (corresponds to “Positive Speech Sentiment”)

sentiment_neg Measure of negative valence of speech ranging from 0-1

sentiment_neu Measure of neutral valence of speech ranging from 0-1

sentiment_overall Measure of overall valence of speech ranging from 0-1

mattr The moving average type token ratio (MATTR) measure of the lexical diversity of speech (corresponds to “Lexical Diversity”)

first_person_percentage Percentage of first-person singular pronoun use

first_person_sentiment_positive Percentage of first-person singular pronoun use multiplied by the score for positive sentiment of speech ranging 0-1

first_person_sentiment_negative Percentage of first-person singular pronoun use multiplied by the score for negative sentiment of speech ranging 0-1

first_person_sentiment_overall Percentage of first-person singular pronoun use multiplied by the score for overall sentiment of speech ranging 0-1

word_repeat_percentage Percentage of words repeated

phrase_repeat_percentage Percentage of phrases repeated

word_coherence Large language model measure of semantic similarity of each word to the immediately preceding word (mean and var)

word_coherence_5 Large language model measure of semantic similarity of each word in a five-word window (mean and var)

word_coherence_10 Large language model measure of semantic similarity of each word in a ten-word window (mean and var)

word_coherence_variability_2-10 Large language model measure of word-to-word variability at k inter-word distances, from two to ten-word distances (mean and var)

*LIWC Variables*

WC Total word count of the text output

Analytic Word use related to achievement

Clout Language related to leadership and status

Authentic Language displaying honesty and genuineness

Tone Degree of positive tone of language

WPS Average number of words per sentence

BigWords Percentage of words with 7 letters or more

Dic Percentage of words captured by the LIWC dictionary

Linguistic Word use with a particular linguistic relevance (e.g. pronouns, determiners and prepositions)

Function Total number of function words

pronoun Overall pronoun use

ppron Personal pronoun use

i First person singular pronoun use

we First person plural pronoun use

you Second person pronoun use

shehe Third person singular pronoun use

they Third person plural pronoun use

ipron Impersonal pronoun use

det Determiner word use (e.g., the, at, that)

article Article use

number Number word use

prep Preposition use

auxverb Auxiliary verb use

adverb Number of adverbs used

conj Number of conjunctions used

negate Number of negation words used

verb Number of verbs used

adj Number of adjectives used

quantity Quantity of words used

Drives Word use related to psychological drive (e.g., work)

Affiliation Word use related to affiliation (e.g., we, our, us)

achieve Word use related to achievement

power Word use related to power

Cognition Word use related to achievement

Allnone All-or-none word use

Cogproc Cognitive process words

Insight Word use related to cognitive insight

Cause Word use related to causation

Discrep Word use identifying discrepancies

tentat Tentative word use

Certitude Word use related to certitude

Differ Words used for the purpose of differentiation

memory Word use related to memory

Affect Word use related to affect

tone_pos Word use indicating a positive tone of speech

tone_neg Word use indicating a negative tone of speech

emotion Word use related to emotion

emo_pos Word use related to positive emotion

emo_neg Word use related to negative emotion

emo_anx Word use related to feeling anxious

emo_anger Word use related to feeling angry

emo_sad Word use related to feeling sad

swear Swear word use

Social Word use related to social processes

socbehav Word use related to social behaviour

prosocial Word use related to prosocial behaviour

polite Word use indicating politeness

conflict Word use indicating conflict

moral Word use related to moralising

comm Word use indicating communication with others

socrefs Word referencing social interaction

family Word use related to family

friend Word use related to friendship

female References to females

male References to males

Culture Word use related to culture

Politic Word use related to politics

Ethnicity Words referencing ethnicity

tech Word use related to technology

Lifestyle Word use related to lifestyle

Leisure Word use related to achievement

Home Word use related to the home

work Word use related to work

money Word use related to money

relig Word use related to religion

Physical Word use related to physical experiences in the body

health Word use related to health

illness Word use related to illness

wellness Word use related to wellness

mental Word use related to mental health

substances Word use referencing drugs (e.g., alcohol, cigarettes)

sexual Word use related to sex and sexuality

food Word use related to food

death Word use related to death

need Word use indicating a need for something

want Word use indicating a want for something

acquire Word use referencing acquisition

lack Word use indicating that something is not possessed

fulfill Word use indicating fulfilment

fatigue Word use indicating the experience of fatigue

reward Word use related to the experience of reward

risk Word use related to the experience of risk

curiosity Word use related to the experience of curiosity

allure Word use related to the experience of being allured by something

Perception Word use related to perceiving something

attention Word use related to attending to something

motion Word use related to motion

space Word use related to the experience of reward

visual Word use related to visual experience

auditory Word use related to auditory experience

feeling Word use related to physically feeling something

time Word use related to the experience of time

focuspast Word use with a focus on the past

focuspresent Word use with a focus on the present

focusfuture Word use with a focus on the future

Conversation Conversational word use (e.g., yeah, okay)

Netspeak Internet language (e.g., lol, u)

Assent Word use indicating assent

Nonflu Non fluent word use (e.g., oh, um)

Filler Filler word use (e.g., wow, you know)

AllPunc All punctuation use

Period Period use

Comma Comma use

QMark Question mark use

Exclam Exclamation use

Apostro Apostrophe use
